# Hinokiflavone induces apoptosis, cell cycle arrest and autophagy in chronic myeloid leukemia cells through MAPK/NF-κB signaling pathway

**DOI:** 10.1186/s12906-022-03580-7

**Published:** 2022-04-06

**Authors:** Xiang Qin, Xi Chen, Ling Guo, Jing Liu, You Yang, Yan Zeng, Cheng Li, Wenjun Liu, Wenzhe Ma

**Affiliations:** 1 State Key Laboratory of Quality Research in Chinese Medicine, Macau University of Science and Technology, Avenida Wai Long, Taipa, 999078 Macau China; 2 Department of Pediatrics, The Affiliated Hospital of Southwest Medical University, Children Hematological Oncology and Birth Defects Laboratory, Sichuan Clinical Research Center for Birth Defects, No. 25, Taiping Street, Jiangyang District, Luzhou, 646000 Sichuan China

**Keywords:** Hinokiflavone, Chronic myeloid leukemia, MAPK, NF-κB, Apoptosis, Autophagy

## Abstract

**Background:**

Chronic myeloid leukemia (CML) is a myeloproliferative tumor originating from hematopoietic stem cells, and resistance to tyrosine kinase inhibitors (TKI) has become a major cause of treatment failure. Alternative drug therapy is one of the important ways to overcome TKI resistance. Hinokiflavone (HF) is a C-O-C type biflavonoid with low toxicity and antitumor activity. This study investigated the antitumor effect and possible mechanisms of HF in CML cells.

**Methods:**

Cell viability was measured by CCK-8 assay. Cell apoptosis and cell cycle distribution were analyzed by flow cytometry. Western blotting was used to assess protein expression levels.

**Results:**

Our results showed that HF significantly inhibited the viability of K562 cells in a concentration- and time-dependent manner and induced G_2_/M phase arrest by up-regulating p21 and down-regulating Cdc2 protein. Furthermore, HF induced caspase-dependent apoptosis by activating JNK/p38 MAPK signaling pathway and inhibiting NF-κB activity. In addition, HF induced autophagy by increasing LC3-II expression and p62 degradation. Pretreatment with CQ, a late autophagy inhibitor, significantly increased the levels of LC3-II and p62 proteins and promoted cell survival.

**Conclusion:**

HF shows a good anti-leukemia effect and is expected to become a potential therapeutic drug for CML.

**Supplementary Information:**

The online version contains supplementary material available at 10.1186/s12906-022-03580-7.

## Introduction

Chronic myeloid leukemia (CML) is a myeloproliferative tumor originating from hematopoietic stem cells, characterized by the formation of the BCR-ABL1 fusion gene [1]. The BCR-ABL1 protein enhances tyrosine kinase activity and contributes to uncontrolled proliferation and apoptosis inhibition [2]. As first-line drugs, tyrosine kinase inhibitors (TKIs) have significantly improved the prognosis of CML, but their clinical application is limited by drug intolerance and drug resistance [3–5]. Therefore, it is of great significance to find novel anti-CML compounds.

**Fig. 1:**
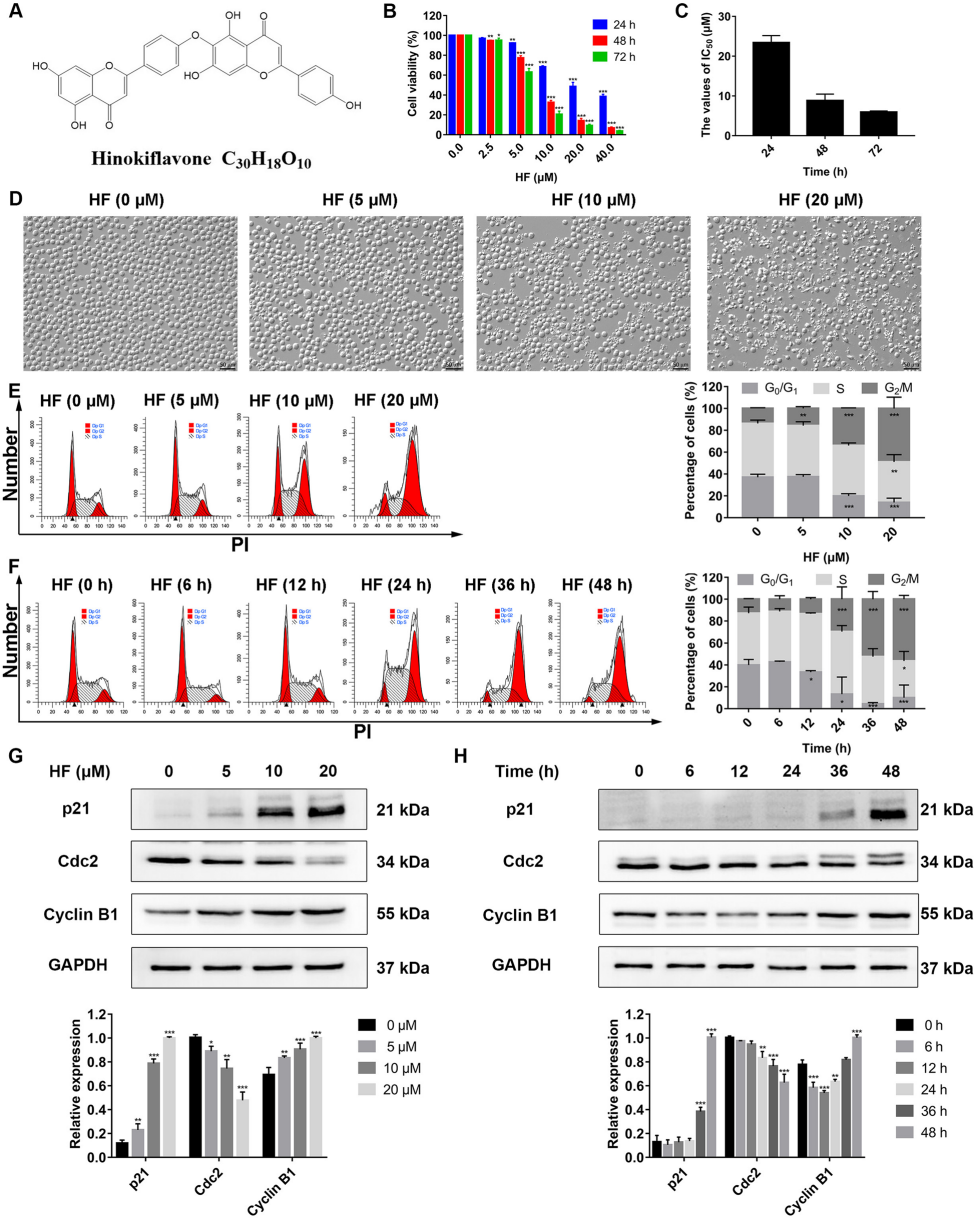
HF inhibits cell proliferation and induces G_2_/M phase arrest in CML cells. **A** The chemical structure of Hinokiflavone (C_30_H_18_O_10_). **B** The cell viability of K562 cells treated with HF (0.0, 2.5, 5.0, 10.0, 20.0, and 40.0 μM) for 24, 48 and 72 h, respectively. **C** The IC_50_ values of HF on K562 cells. **D** The morphology changes of cells treated with HF for 48 h were observed by optical microscopy (× 200, scale bar = 50 μm). **E** Cell cycle distribution of K562 cells treated with HF (0, 5, 10 and 20 μM) for 48 h. **F** Cell cycle distribution of K562 cells treated with 10 μM HF at different time (0, 6, 12, 24, 36 and 48 h). **G**, **H** The expression levels of p21, Cdc2 and Cyclin B1 were analyzed by western blotting after treatment with different concentrations of HF (0, 5, 10 and 20 μM) and different times of 10 μM HF. *P < 0.05, **P < 0.01, ***P < 0.001 vs the control group

Nature products are an essential resource of antitumor drugs [6]. Biflavonoids are polyphenolic compounds widely distributed in plants [7]. Hinokiflavone (HF) (chemical structure showed in Fig. 1A) is a C-O-C type biflavonoid isolated from *Selaginella tamarisina* (*P. Beauv.*) spring and other plants [7–9]. It has diverse pharmacological activities, including anti-inflammation [10], antivirus [11] and antioxidant [12]. In addition, HF modulates pre-mRNA splicing and inhibits sentrin-specific protease 1 (SENP1) [13]. Recently, a large number of studies reported the antitumor activities of HF in solid tumors by inducing apoptosis, blocking the cell cycle, and inhibiting invasion and metastasis [14–17]. Nonetheless, the effect of HF on leukemia and the underlining mechanism have not been comprehensively evaluated.

Mitogen-activated protein kinase (MAPK) signaling pathways, including extracellular signal regulated protein kinase (ERK), p38 and c-Jun N-terminal kinase (JNK), are important ways to transfer exogenous stimulus into cells [18]. Dysregulated MAPK signaling is one of the major factors contributing to CML pathogenesis [19] and resistance to antitumor drugs [20]. Nuclear factor-kappa B (NF-κB) is a crucial regulator in the malignant transformation and survival of leukemia cells [21, 22]. In addition, accumulating evidence indicates that the mutual crosstalk exists between MAPK and NF-κB signaling pathways [23–25], the most important one of which is mediated by the Gadd45 family of proteins. Gadd45 proteins are a group of critical signal sensor involved in the regulation of multiple cell functions by connecting upstream receptor module transcription NF-κB and transcription regulation module MAPK. However, the correlation between NF-κB and MAPK is not only a simple upstream and downstream regulation, but also linked through activation, collaboration, crosstalk, feedback and other mechanisms [26]. In this study, we evaluated the antitumor activities of HF and its effect on MAPK and NF-κB pathways in human CML cells.

## Materials and methods

### Reagents and cell lines

Hinokiflavone (HF) with purity > 97% was purchased from Chengdu Herbpurify CO., LTD (Chengdu, China). SP600125, SB203580, U0126, 3-Methyladenine (3-MA) and Chloroquine phosphate (CQ) were obtained from MedChemExpress (New Jersey, USA). p-p38 (4511S), p38 (8690S), p-ERK1/2 (9101S), ERK1/2 (9102S), p-MEK1/2 (9154S), MEK1/2 (9122S), p-JNK (9251S), JNK (9252S), p-NF-κB p65 (3033S), NF-κB p65 (8242S), Cyclin B1 (12231S), Cdc2 (9116S), p21 (2947S), Cleaved caspase-3 (9664S), Cleaved caspase-9 (7237S), Cleaved PARP (9546S), and LC3 (12741S) were provided by Cell Signaling Technology (Beverly, MA, USA). p62 (ab82645) was provided by Abcam (Cambridge, UK). GAPDH (10494–1-AP) was provided by Proteintech Group (Wuhan, China). Anti-rabbit (A0208) and anti-mouse (A0216) secondary antibodies were purchased from Beyotime Biotechnology (Shanghai, China).

### Cell culture

K562 cell line was purchased from ZhongqiaoXinzhou Biotechnology CO., LTD (Shanghai, China). Cells were cultured in RPMI-1640 medium (Gibco, USA) supplemented with 10% fetal bovine serum (Biological Industries, Kibbutz Beit Haemek, Israel), 100 U/mL penicillin and 100 μg/mL streptomycin (Solarbio, Shanghai, China), and stored at 37 °C in a humidified atmosphere with 5% CO_2_.

### Cell viability assay

The cell viability was measured by cell counting kit 8 (CCK-8) assay (Dojindo, Kumamoto, Japan). Logarithmic growthing cells were inoculated into 96-well plates at 5.0 × 10^3^ cells/well, and treated with HF (0.0, 2.5, 5.0, 10.0, 20.0, and 40.0 μM) for 24, 48 and 72 h, respectively. Then, 10 μL CCK-8 was added into each well. After incubated for 4 h at 37 °C, the optical density (OD) was measured at 450 nm with a spectrophotometer (Beckman, Fullerton, CA, USA). Cell viability was calculated according to the following formula: cell viability (%) = [(the absorbance of experimental group - the absorbance of blank group) / (the absorbance of untreated group - the absorbance of blank group)] × 100%.

### Hoechst 33258 staining

The cells were seeded in a 6-well plate and exposed to HF for 48 h, then washed with PBS and stained with Hoechst 33258 solution (Solarbio, Shanghai, China) for 15 min at 37 °C. Finally, the nuclear morphology was photographed by a fluorescence microscopy (Olympus, Tokyo, Japan).

### Cell apoptosis analysis

Cell apoptosis was detected by FITC Annexin V Apoptosis Detection Kit I (BD Pharmingen™, San Diego, CA, USA). After treated with different concentrations of HF for 48 h, the cells were harvested and washed twice with precooled PBS and resuspended in 1 × binding buffer at a density of 1 × 10^5^ cells/100 μL. Then, 5 μL FITC Annexin V and 5 μL PI were added, gently vortexed and incubated for 15 min at room temperature in the dark. Finally, 400 μL 1 × binding buffer was added into each tube, and the cells were analyzed by flow cytometer (BD Biosciences, Franklin Lakes, NJ, USA).

### Cell cycle distribution

Cell cycle distribution was measured by cell cycle detection kit (KeyGEN, Nanjing, China). The cells were collected and washed twice with PBS, and fixed with 70% precooled ethanol overnight at 4 °C. Washed once with PBS and centrifuged at 1000 rpm for 3 min. Then, the cells were resuspended in propidium iodide (PI) and RNase A and incubated for 30 min at room temperature in the dark. Finally, cell cycle distribution was analyzed by flow cytometer (BD Biosciences, Franklin Lakes, NJ, USA).

### Western blotting

The cells were harvested, washed twice with cold PBS, and lysed in lysis buffer (Beyotime, Shanghai, China). Protein concentration was detected by bicinchoninic acid protein assay kit (BioTeke, Beijing, China). The proteins were separated by SDS-PAGE (Solarbio, Shanghai, China) with equal quantities of protein, and transferred to polyvinylidene fluoride membranes (Millipore, Billerica, MA, USA). The membranes incubated with primary antibodies overnight at 4 °C. Washed and incubated with the appropriate secondary antibodies conjugated with horseradish peroxidase (Beyotime, Shanghai, China). Finally, the protein bands were detected by enhanced chemiluminescence (ECL) system (Vilber, Torcy, France). GAPDH was used as the loading control. The optical density of the bands was analyzed by Image J software, and the relative expression level of the target protein was displayed by column chart.

### Statistical analysis

Statistical analysis was performed using the software SPSS 20.0. All experiments were repeated three times independently, and the data were expressed as mean ± standard deviation (SD). Statistical analyses were analyzed by unpaired two-tailed Student’s t-test or one-way analysis of variance (ANOVA). *P* < 0.05 was considered statistically significant.

## Results

### HF inhibits CML cells proliferation

CCK-8 assay was performed to detect the effects of HF on the proliferation of K562 cells. The results showed that HF significantly inhibited the viability of K562 cells in a concentration- and time-dependent manner (Fig. 1B), and the IC_50_ values were 23.38 ± 1.78 μM, 8.84 ± 1.62 μM, and 5.93 ± 0.28 μM at 24, 48, and 72 h, respectively (Fig. 1C). Moreover, cell morphology was observed by microscopy. The cells became irregular, shrunk and disintegrated with the increase of HF concentration (Fig. 1D). Altogether, these results indicate that HF has an anti-proliferative effect against CML cells.

### HF induces G_2_/M phase arrest in K562 cells

To investigate the mechanism of HF inhibited proliferation of K562 cells, we analyzed cell cycle distribution by flow cytometry. As shown in Fig. 1E and F, the proportion of cells in G_2_/M phase increased in a concentration- and time-dependent manner. Then, the expression of cell cycle-related proteins was detected by western blotting (Fig. 1G and H). HF increased the protein levels of p21 and decreased Cdc2 in a concentration- and time-dependent manner. Taken together, these results suggest that HF induces G_2_/M phase arrest by regulating the p21/Cdc2 signaling pathway.

### HF induces caspase-mediated apoptosis in K562 cells

**Fig. 2:**
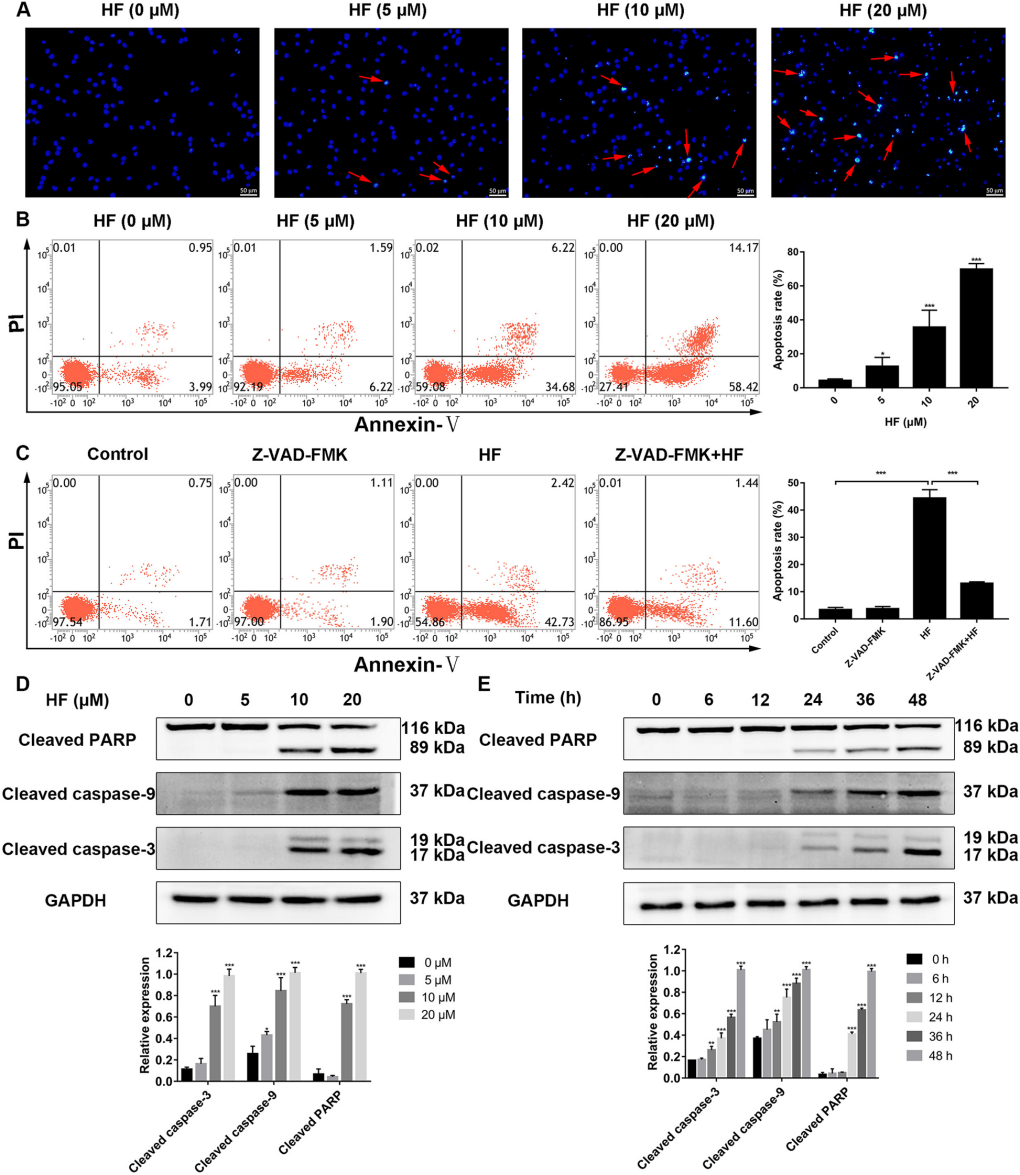
HF induces caspase-dependent apoptosis in K562 cells. **A** The nuclear changes were determined by Hoechst 33258 staining and photographed by fluorescence microscopy (× 200, scale bar = 50 μm). **B** The proportion of apoptotic cells treated with HF (0, 5, 10 and 20 μM) for 48 h was analyzed by flow cytometry. **C** The apoptosis of K562 cells pre-treated with 40 μM Z-VAD-FMK for 1 h before exposed to 10 μM HF for 48 h. **D**, **E** The expression levels of cleaved caspase-3, cleaved caspase-9 and cleaved PARP were detected by western blotting after treatment with different concentrations of HF (0, 5, 10 and 20 μM) and different times of 10 μM HF. *P < 0.05, **P < 0.01, ***P < 0.001 vs the control group

Induction of apoptosis is another antitumor mechanism of natural compounds. So, we evaluated the effect of HF on apoptosis in K562 cells. Hoechst 33258 staining detected the nuclear changes of K562 cells treated with HF for 48 h. The number and intensity of cells with bright-blue fluorescence increased significantly, and the cells shrank, chromatin condensed and nuclear fragmentation (Fig. 2A). Flow cytometry analysis showed that HF increased the proportion of apoptotic cells in a concentration-dependent manner (Fig. 2B). Pretreatment with Z-VAD-FMK, a pan-caspase inhibitor, rescued K562 cells from HF induced apoptotic cell death (Fig. 2C). In addition, the apoptosis-related proteins were analyzed by western blotting. The levels of cleaved caspase-9, cleaved caspase-3 and cleaved PARP were up-regulated in a concentration- and time-dependent manner (Fig. 2D and E). In conclusion, these results indicate that HF induces caspase-dependent apoptosis in CML cells.

### HF induces apoptosis through MAPK / NF-κB signaling pathway

**Fig. 3:**
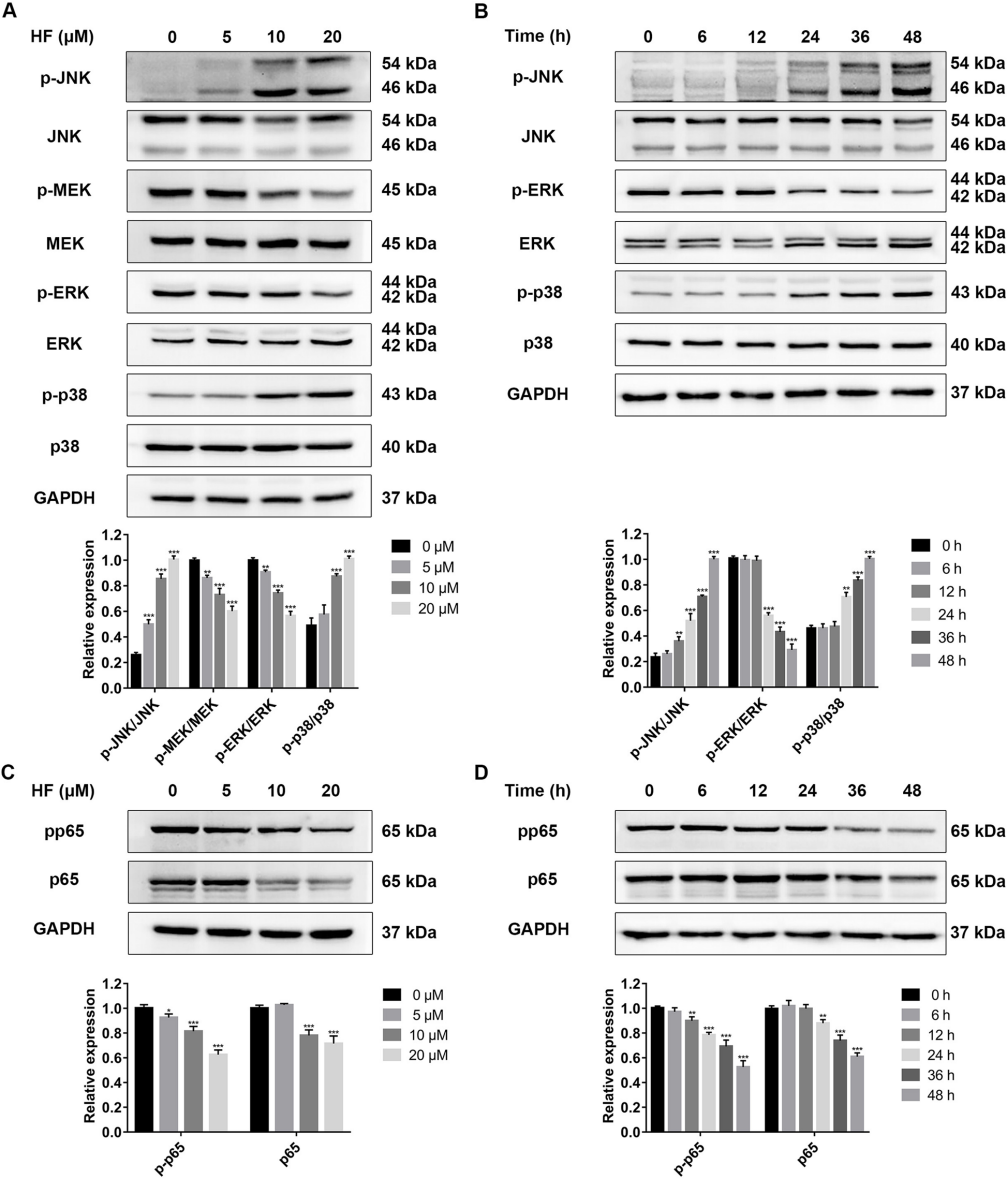
Effects of HF on MAPK and NF-κB signaling pathways. **A**, **C** The expression levels of MAPK and NF-κB pathway related proteins in K562 cells treated with HF for 48 h. **B**, **D** The expression levels of MAPK and NF-κB pathway related proteins in K562 cells treated with 10 μM HF at different time. *P < 0.05, **P < 0.01, ***P < 0.001 vs the control group

**Fig. 4:**
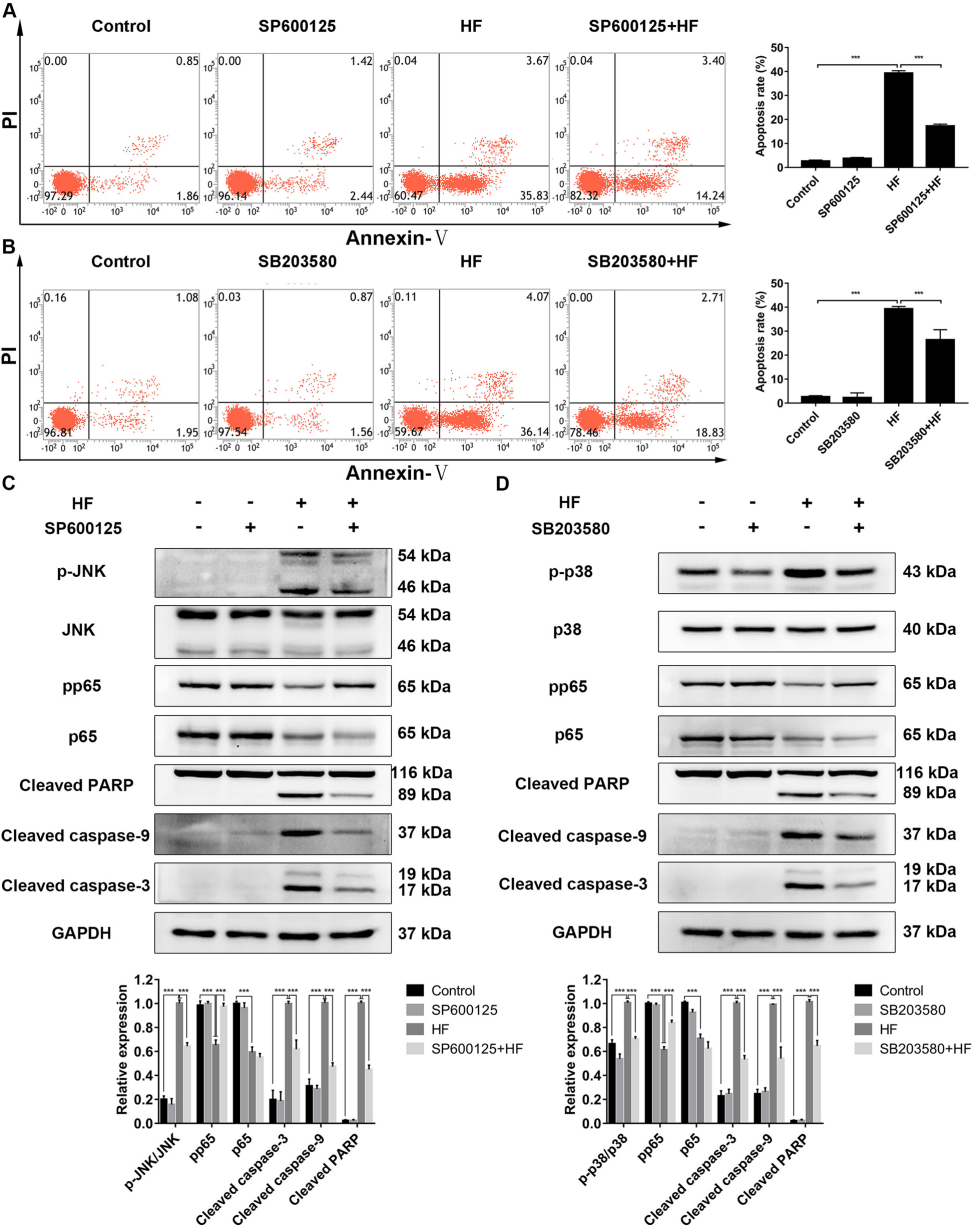
HF induces apoptosis by activating JNK / p38 MAPK signaling pathway and inhibiting NF-κB activity in K562 cells. **A**, **B** Flow cytometry was used to analyze the apoptosis of K562 cells pre-treated with 10 μM SP600125 or 10 μM SB203580 for 1 h before exposed to 10 μM HF for 48 h. **C**, **D** The expression levels of proteins in K562 cells pre-treated with 10 μM SP600125 or 10 μM SB203580 for 1 h before exposed to 10 μM HF for 48 h. *P < 0.05, **P < 0.01, ***P < 0.001

To investigate whether MAPK and NF-κB signaling pathways mediated the anti-leukemia effect of HF, we analyzed the relevant proteins by western blotting. It was showed that HF increased the phosphorylation levels of p38 and JNK while decreasing ERK phosphorylation levels in a concentration- and time-dependent manner (Fig. 3A and B). Also, the protein levels of total p65 and phosphorylated p65 were significantly down-regulated (Fig. 3C and D). To confirm the involvement of MAPK and NF-κB signaling pathways in HF-induced apoptosis, we pre-treated K562 cells with 10 μM SB203580 (p38 inhibitor) or 10 μM SP600125 (JNK inhibitor) for 1 h before the treatment with 10 μM HF for 48 h. Flow cytometry analysis revealed that SP600125 and SB203580 pretreatment significantly reduced the proportion of apoptotic cells (Fig. 4A and B). Moreover, SP600125 pretreatment inhibited JNK signaling pathway, restored the expression of phosphorylated p65, and decreased the levels of cleaved caspase-9, cleaved caspase-3 and cleaved PARP protein (Fig. 4C). Similar results were obtained with SB203580 pretreatment (Fig. 4D). These results suggest that HF induces apoptosis by activating JNK/p38 MAPK signaling pathway and inhibiting NF-κB activity in CML.

### HF induces autophagy in K562 cells

**Fig. 5:**
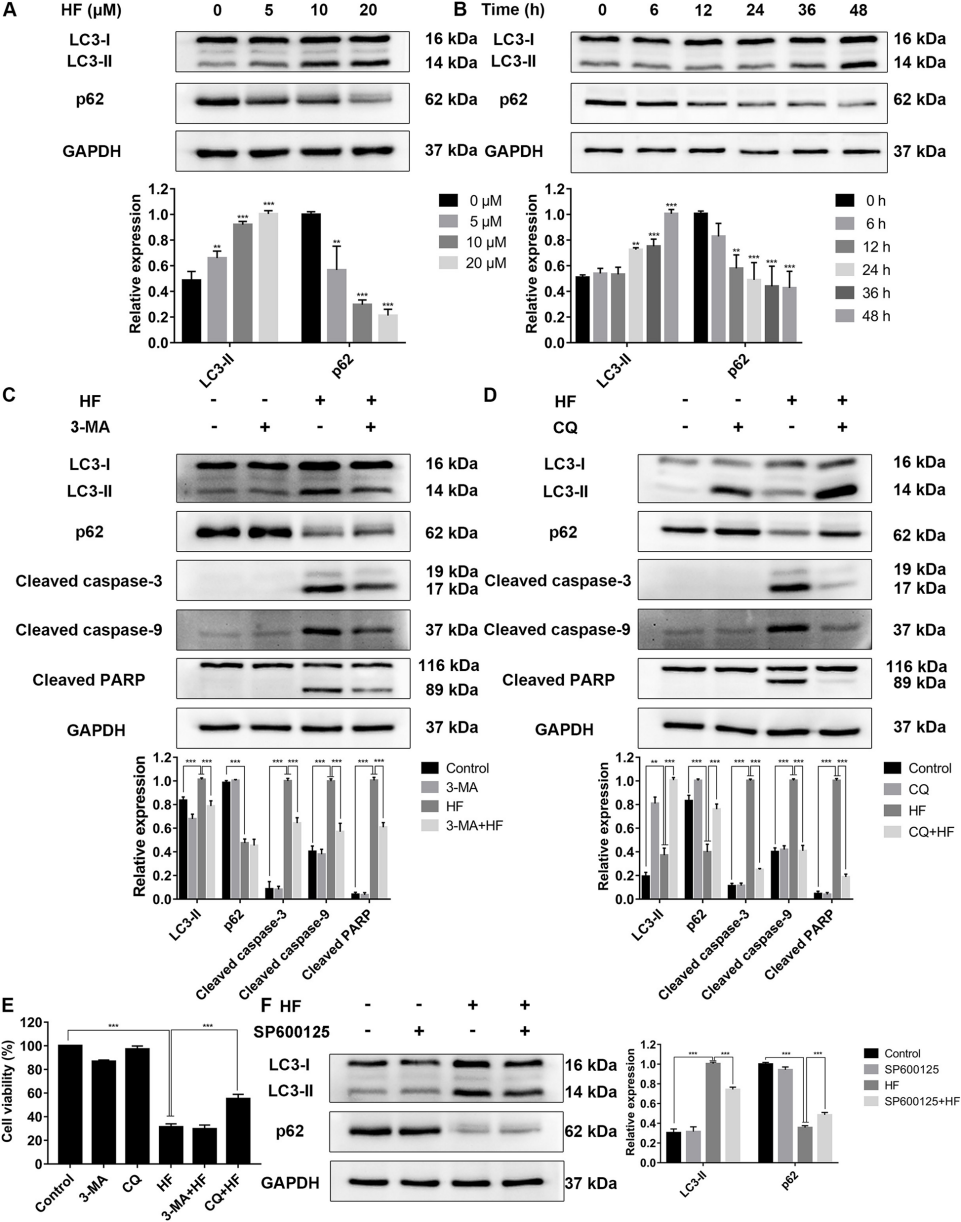
HF induces autophagy in K562 cells. **A**, **B** The expression levels of LC3-II and p62 proteins in K562 cells after treatment with different concentrations of HF (0, 5, 10 and 20 μM) and different times of 10 μM HF. **C**, **D** The expression levels of proteins in K562 cells pre-treated with 1 mM 3-MA or 5 μM CQ for 1 h before exposed to 10 μM HF for 48 h. **E** The cell viability of K562 cells measured by CCK-8 assay. **F** The expression levels of LC3-II and p62 in K562 cells pre-treated with 10 μM SP600125 for 1 h before exposed to 10 μM HF for 48 h. *P < 0.05, **P < 0.01, ***P < 0.001

This study also investigated the role of autophagy in the anti-proliferation of HF. The data showed that HF increased autophagy protein LC3-II and decreased the expression of p62 in a concentration- and time-dependent manner (Fig. 5A and B). Then, we used autophagy inhibitors to monitor autophagy flux. Pretreatment with 3-MA, an early autophagy inhibitor, decreased the expression of LC3-II and caspase-related proteins but did not affect cell viability (Fig. 5C and E). Pretreatment with CQ, a late autophagy inhibitor, increased the levels of LC3-II and p62 and rescued cell viability, but decreased the expression of caspase-related proteins (Fig. 5D and E). Therefore, HF inhibits the proliferation of K562 cells by activating autophagy. In addition, our study showed that the expression of LC3-II was decreased by SP600125 pretreatment (Fig. 5F). However, the normal concentration of SB203580 (10 μM) promoted the expression of LC3-II and p62 protein in K562 cells and interfered with the monitoring of autophagy flux (Fig. S1). In summary, these results suggest that HF induces autophagy, which may be related to the activation of JNK signaling pathway.

## Discussion

Biflavonoids exhibit multiple pharmacological activities in microbial diseases, diabetes, cognitive disorders, and cancer progression and metastasis [7–9]. HF is a C–O–C type biflavonoid (Fig. 1A). It has been shown selective cytotoxicity against tumor cells over normal cells [14–16]. This study expanded its antitumor activities on K562 cells and elucidated the anti-leukemia mechanisms.

In the present study, CCK-8 assay showed that HF inhibited the proliferation of K562 cells in a concentration- and time-dependent manner (Fig. 1B). Similar effects of HF have been observed in other tumor cells, such as hepatocellular carcinoma, colorectal cancer, and melanoma [14–16]. These results suggested that HF possessed significant anti-proliferative activity against various tumor cells.

Disturbance of cell cycle regulation is an important cause of tumorigenesis, and targeted cell cycle therapy is considered a promising anti-cancer strategy [26]. In this study, flow cytometry analysis showed that HF induced G_2_/M phase arrest (Fig. 1D and E). p21 is an important member of the Cip/Kip family of cyclin-dependent kinase (CDK) inhibitors, which regulates cell cycle progression by binding and inactivating CDKs complex [27]. Cyclin B1/Cdc2 complex is a key regulator of mitotic entry, and its activity determines whether cells enter mitosis or arrest at G_2_ phase [28]. We further studied the expression of related proteins in G_2_/M phase, and the results showed that HF enhanced the expression of p21 protein and suppressed the expression of Cdc2 protein (Fig. 1F and G). It has been reported that p21 can inactivate Cyclin B1/Cdc2 complex and lead to G_2_/M phase arrest [29]. Thus, these results demonstrate that HF induces G_2_/M phase arrest by modulating the activity of p21 and Cdc2 in K562 cells. However, HF also induced G_0_/G_1_ phase and S-phase arrest in hepatocellular carcinoma and melanoma cells, respectively [14, 16]. Taken together, HF plays an important role in the regulation of tumor cell cycle progression.

Induction of apoptosis is the common mechanism of natural compounds with antitumor activities [6]. HF can induce intracellular reactive oxygen species (ROS), promote the release of pro-apoptotic factors, and recruit and activate caspase 9, thereby initiating the caspase cascade to mediate apoptosis [14–17]. In this study, HF induced mitochondrial-mediated apoptosis by increasing cleaved caspase-9, cleaved caspase-3 and cleaved PARP in K562 cells (Fig. 2A-E). MAPK cascades are important intracellular signal transduction pathways, which participate in cell apoptosis, proliferation and differentiation by regulating the activity of the downstream transcription factors [18]. NF-κB transcription factor is a key regulator of cell survival, regulating apoptosis, autophagy and necrosis of tumor cells [22]. Therefore, this study investigated the role of MAPK and NF-κB signaling pathways in HF-induced apoptosis. The data showed that HF activated JNK and p38 MAPK signaling pathways, and inhibited MEK/ERK and NF-κB signaling pathway (Fig. 3A-D). Interestingly, inhibiting the JNK and p38 MAPK pathways by SP600125 and SB203580, respectively, restored NF-κB activity and reversed HF-induced apoptosis (Fig. 4A-D). Our results suggest that HF induces caspase-dependent apoptosis through JNK/p38 MAPK/NF-κB signaling pathway in K562 cells. Similar results have been found for HF in hepatocellular carcinoma, HF induces apoptosis through activating JNK signaling pathway and inhibiting NF-κB activity. However, the activation of p38 MAPK signaling pathway had no significant effect on HF-induced apoptosis in hepatocellular carcinoma cells [14].

Natural biflavonoids inhibit tumorigenesis by regulating the crosstalk between apoptosis and autophagy [6, 9]. Currently, the role of autophagy in HF-induced cell death has not been reported. Therefore, our study first analyzed the expression of the autophagy marker protein LC3. LC3-I is conjugated with phosphatidylethanolamine to form LC3-II, which is attached to the autophagosome membrane, so the level of LC3-II reflects the number of autophagosomes to a certain extent [30]. The results showed that the levels of LC3-II increased in a concentration- and time-dependent manner after HF intervention (Fig. 5A and B). p62 acts as a link between LC3 and the ubiquitinated substrate and is then integrated into autophagosome and degraded in the autophagolysosome [30]. Therefore, the expression level of p62 also reflects autophagy activity. In this study, HF significantly reduced p62 expression in a concentration- and time-dependent manner (Fig. 5A and B). We further evaluated the autophagy flux using autophagy late inhibitors. CQ inhibits the fusion of autophagosomes and lysosomes, leading to the accumulation of autophagosomes [31]. Our data showed that CQ treatment significantly increased the levels of LC3-II and p62, inhibited the expression of pro-apoptotic proteins and promoted cell survival (Fig. 5D and E). These results suggest that HF can induce cell death by activating autophagy in K562 cells. It is well known that JNK and p38 MAPK signaling pathways are involved in the regulation of autophagy [6]. Our data showed that inhibition of JNK signaling pathway by SP600125 attenuated HF-induced LC3-II expression (Fig. 5F). These results indicate that HF can induce autophagy by activating JNK signaling pathway. However, SB203580 (10 μM) significantly promoted the expression of LC3-II and p62 proteins (Fig. S1), and seriously interfered with the autophagy flux in a p38-independent manner. In addition, several pieces of evidence showed that low concentrations of pyridylimidazole inhibitors such as SB202190 have an effect on autophagy in some cells, but whether SB203580 affects autophagy remains unclear [32, 33]. Therefore, other pharmacological tools are needed to clarify the relationship between the p38 MAPK pathway and HF-induced autophagy.

**Fig. 6:**
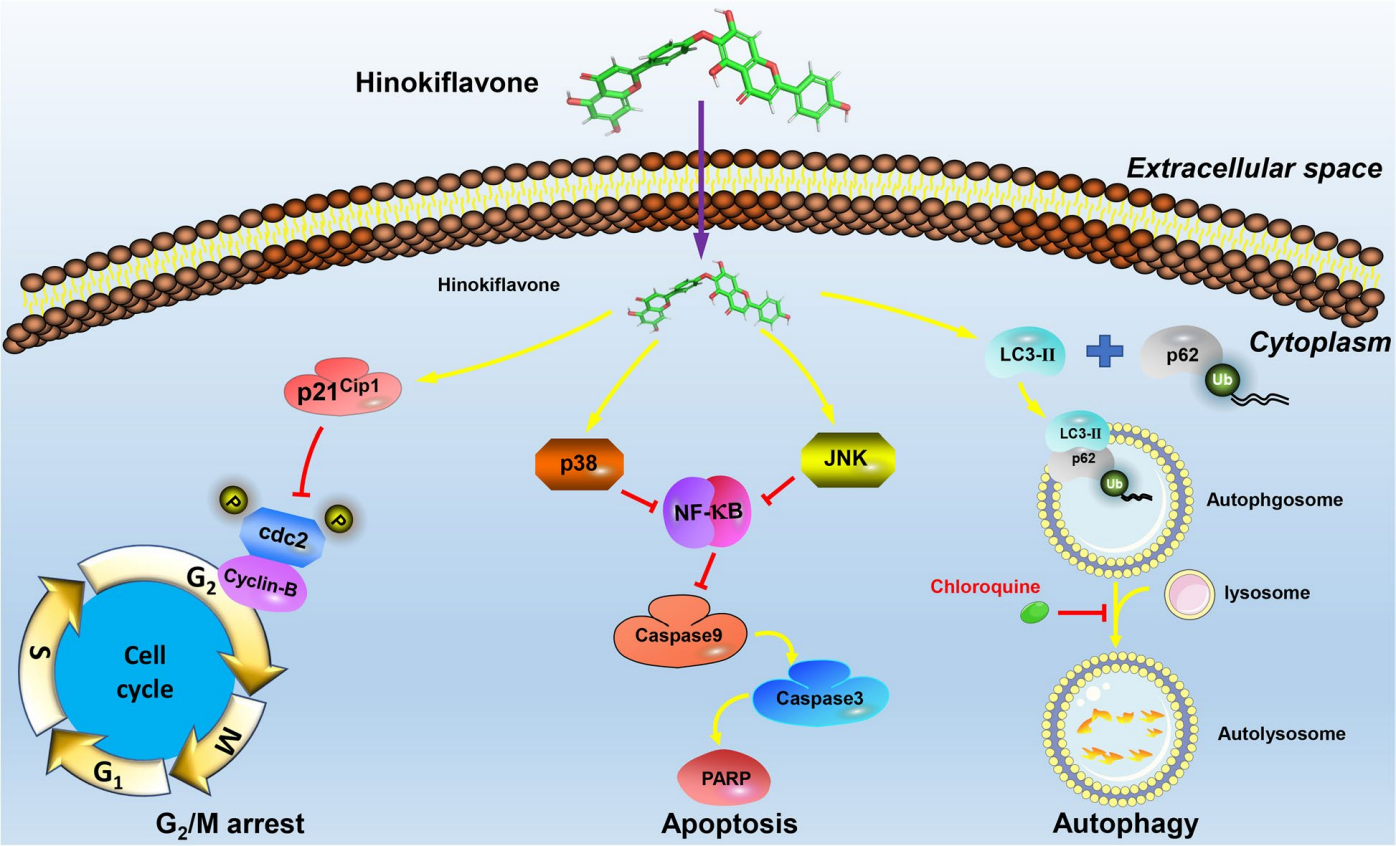
The mechanisms of HF against CML

In summary, HF induced caspase-mediated apoptosis, G_2_/M phase arrest and autophagy to inhibit the proliferation of CML cells through MAPK/NF-κB signaling pathway (Fig. 6). This study demonstrated that HF might be a potential drug for the treatment of CML. However, this study only used one CML cell line with certain limitations. Therefore, further experiments in more cell lines and animals will help to evaluate the future research prospects of HF.

## Supplementary Information

**Additional file 1: Fig. S1.** The effect of p38 MAPK signaling pathway on HF-induced autophagy in K562 cells.

## Data Availability

The datasets used and/or analysed during the current study are available from the corresponding authors on reasonable request.

## Abbreviations

CML: Chronic myeloid leukemia
TKI: Tyrosine kinase inhibitors
HF: Hinokiflavone
CCK-8: Cell counting kit-8
SENP1: Sentrin-specific protease 1
MAPK: Mitogen-activated protein kinase
ERK: Extracellular signal regulated protein kinase
JNK: c-Jun N-terminal kinase
NF-κB: Nuclear factor-kappa B
3-MA: 3-Methyladenine
CQ: Chloroquine phosphate
