# Supplementary material for: Hinokiflavone induces apoptosis, cell cycle arrest and autophagy in chronic myeloid leukemia cells through MAPK/NF-κB signaling pathway
Source: BMC Complement Med Ther. 2022 Apr 6;22:100. doi: 10.1186/s12906-022-03580-7 (PMC8988348; doi:10.1186/s12906-022-03580-7)
Supplement: Supplementary file 1 — Additional file 1: Fig. S1. The effect of p38 MAPK signaling pathway on HF-induced autophagy in K562 cells. [file 12906_2022_3580_MOESM1_ESM.doc]

**Supplementary Information**

**Fig. S1 The effect of p38 MAPK signaling pathway on HF-induced autophagy in K562 cells.**


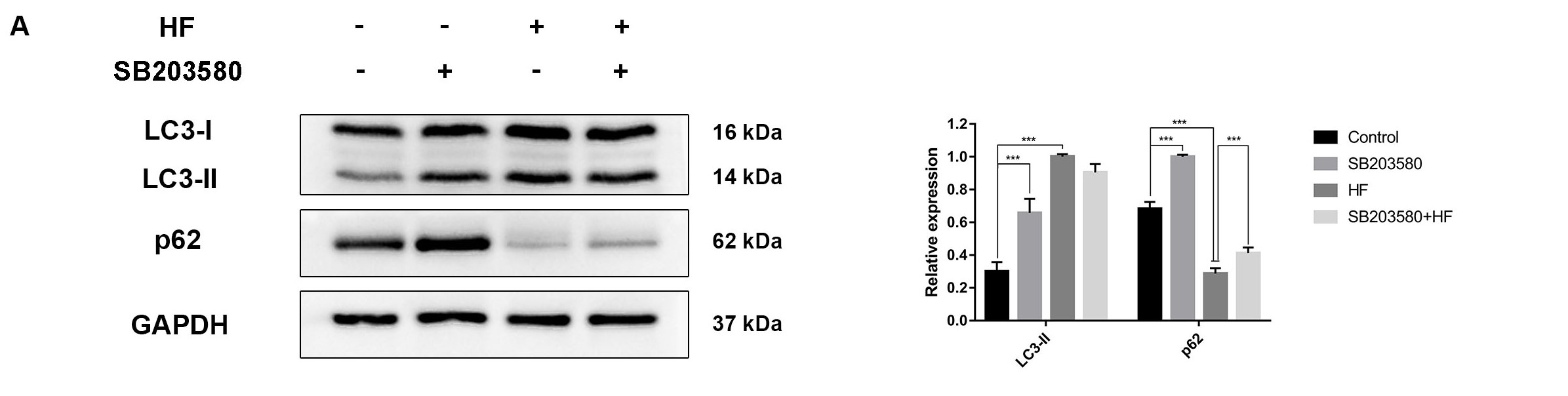


**Fig. S1 The effect of p38 MAPK signaling pathway on HF-induced autophagy in K562 cells.** (**A**) The expression levels of LC3-II and p62 in K562 cells pre-treated with 10 µM SB203580 for 1 h before exposed to 10 µM HF for 48 h. ****P* < 0.001.
